# Developmental and Molecular Changes Underlying the Vernalization-Induced Transition to Flowering in *Aquilegia coerulea* (James)

**DOI:** 10.3390/genes10100734

**Published:** 2019-09-22

**Authors:** Bharti Sharma, Timothy A. Batz, Rakesh Kaundal, Elena M. Kramer, Uriah R. Sanders, Valerie J. Mellano, Naveen Duhan, Rousselene B. Larson

**Affiliations:** 1 Department of Biological Sciences, California State Polytechnic University Pomona, CA 91768, USA; ursanders@cpp.edu; 2 Plant Science Department, California State Polytechnic University Pomona, CA 91768, USA; tbatz@purdue.edu (T.A.B.); vjmellano@cpp.edu (V.J.M.); 3 Department of Plants, Soils and Climate / Center for Integrated BioSystems, Utah State University, Logan UT 84322, USA; rkaundal@usu.edu (R.K.); naveen.duhan@aggiemail.usu.edu (N.D.); 4 Bioinformatics Facility, Center for Integrated BioSystems, Utah State University, Logan UT 84322, USA; rousselene.jones@usu.edu; 5 Department of Organismic and Evolutionary Biology, Harvard University, Cambridge MA 02138, USA; ekramer@oeb.harvard.edu

**Keywords:** vernalization, *FLOWERING LOCUS T*, inflorescence meristem, floral meristem, flowering, genetic pathways, *Aquilegia*

## Abstract

Reproductive success in plants is dependent on many factors but the precise timing of flowering is certainly among the most crucial. Perennial plants often have a vernalization or over-wintering requirement in order to successfully flower in the spring. The shoot apical meristem undergoes drastic developmental and molecular changes as it transitions into inflorescence meristem (IM) identity, which then gives rise to floral meristems (FMs). In this study, we have examined the developmental and gene expression changes underlying the transition from the vegetative to reproductive phases in the basal eudicot *Aquilegia coerulea*, which has evolved a vernalization response independently relative to other established model systems. Results from both our histology and scanning electron studies demonstrate that developmental changes in the meristem occur gradually during the third and fourth weeks of vernalization. Based on RNAseq data and cluster analysis, several known flowering time loci, including *AqFT* and *AqFL1*, exhibit dramatic changes in expression during the fourth week. Further consideration of candidate gene homologs as well as unexpected loci of interest creates a framework in which we can begin to explore the genetic basis of the flowering time transition in *Aquilegia*.

## 1. Introduction

Flowers are a synapomorphy of the angiosperms that have been a driving force in their rapid diversification into ~350,000+ species over the last ~140 million years [1]. For the reproductive success of plants, accurately timing the transitioning from the vegetative state to flowering is critical. Flowering at a time when the season favors both pollination and seed dispersal is key for successful outcrossing and reproductive success [2]. The careful orchestration and integration of exogenous and endogenous signals requires this process to be highly regulated at the genetic level [3]. Besides the maturity of the plant, the effects of the photoperiod, temperature, and hormones play important roles in the transition to flowering. In the context of temperature, vernalization or exposure to the prolonged cold is necessary for plants such as winter annuals to flower [4]. Genetic mechanisms are wired in such a way that plants can distinguish between proper vernalization in contrast to short cold snaps. This is very important for the plants because premature flowering in the winter season itself can take a toll on reproductive success or other terms of fitness. Appropriate vernalization triggers genetic and developmental changes in the shoot apical meristem (SAM) resulting in its transition to inflorescence meristem (IM) identity, which then gives rise to either terminal or lateral flower meristems (FMs).

The genetic mechanisms underlying vernalization have been studied in several plant models, although the core eudicot *Arabidopsis thaliana* remains the best understood at the molecular level. Flowering in *A. thaliana* is controlled by multiple exogenous and endogenous pathways that integrate both biotic and abiotic signals [5]. Our understanding of the vernalization pathway has been facilitated by the fact that not all *A. thaliana* accessions require vernalization [6]. Much of this variation is due to the natural segregation of non-functional alleles of the MADS-box transcription factor *FLOWERING LOCUS C (FLC)*, which acts as a flowering repressor that keeps plants in a vegetative state in the absence of vernalization [7,8]. Prior to vernalization, *FLC* is highly expressed in vegetative tissues, leaves, and both the root and shoot meristems, but, as vernalization proceeds, *FLC* expression continuously decreases [8,9]. *FLC* remains down-regulated following vernalization thanks to epigenetic silencing, but its expression is reactivated in seeds, which allows the next generation to re-establish the vernalization requirement [9,10]. Down-regulation of *FLC* relieves the repression of the floral promoter *FLOWERING LOCUS T (FT),* which results in the further induction of a broad suite of flowering-associated genes, including *SUPPRESSOR OF CONSTANS 1 (SOC1)* and *AGL24* [11,12]. These loci promote the transition of the SAM to IM identity, which, in turn, leads to the activation of FM identity. This pathway is best represented by the transcription factors *LEAFY* (*LFY*) and *APETALA1* (*AP1*), which are directly activated by *FT* and also provides positive feedback for each other [13] As noted above, several accessions of *Arabidopsis* do not require vernalization, and these are associated with nonfunctional alleles of *FLC* and another locus, *FRIGIDA* (*FRI)*, which upregulates *FLC* before vernalization [14]. While this *FLC*-dependent vernalization response is clearly conserved across the Brassicaceae [15], it appears that different genetic programs are responsible for vernalization in models such as wheat [16] or beet [17]. Yet, they all converge on homologs of *FT* as the primary activator of flowering.

**Figure 1:**
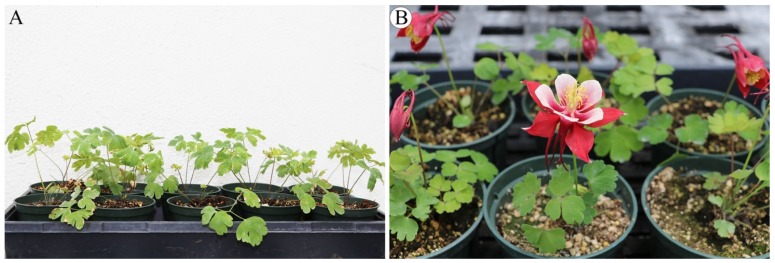
*Aquilegia coerulea* ‘Origami’ plants. (**A**) Vegetative phase, pre-vernalization. (**B**) Flowering phase, post-vernalization.

To broaden the understanding of flowering time genetics in taxa requiring vernalization, we have used *Aquilegia coerulea* as our model system in this study. The genus *Aquilegia* is a basal eudicot with ~70 species that have acquired diverse habitats from Eurasia to North America [18]. It has been best studied for its interesting floral morphology, which includes spurred petals—a key innovation related to the radiation of the genus—as well as petaloid sepals, and the novel staminodes [19,20,21]. The *A. coerulea* genome has been sequenced and functional genetic studies using virus-induced gene silencing has significantly elucidated the developmental genetic basis of these floral traits [22,23,24]. *A. coerulea* is also a promising model for the investigation of flowering time, particularly vernalization [20] (Figure 1). Previous studies of the control of flowering time in the species *A. formosa* found that it does not have a photoperiod response but strongly responds to cold treatment [25]. Homologs of the loci associated with IM and FM identity in *A. thaliana* were expressed in patterns consistent with potentially conserved functions, but most of the flowering time-related genes had divergent gene expression patterns and no homolog of *FLC* has been identified in the genome [25].

We had two main aims for this study. First, we sought to understand how the developmental transitions in the meristem from SAM to IM and then to FM occur over the course of vernalization, which has never been directly examined in *A. coerulea* ‘Origami’, which is the main functional model in *Aquilegia*. The second aim was to develop a transcriptomic resource that provides a more global picture of the reproductive transition and can help us expand the current candidate gene list for functional testing in the future. We are particularly interested in identifying other potential repressors of *FT* that could control transition to flowering in the absence of *FLC*. Adding *Aquilegia* to our repertoire of flowering time models will help us more broadly to understand how these genetic pathways have evolved in plants outside core eudicots and grasses. The current study has uncovered the exact timing of the floral transition in ‘Origami’ and highlighted potential floral repressors, including paralogs of *FT* and *AGL24*. 

## 2. Materials and Methods 

### 2.1. Plant Growth and Histology 

Seeds for *Aquilegia coerulea* ‘Origami Red and White’ were obtained from Swallowtail Seeds (Santa Rosa, CA, USA)*. A. coerulea* plants were grown to an eight-leaf stage in the Cal Poly Pomona greenhouse. Plants were vernalized at 6 °C for 4 weeks (Percival E41VL, Perry, IA, USA). For each time point (pre-vernalization, weeks 1, 2, 3, 4, and post vernalization weeks 5, 6, 7, and 8), apical meristems were dissected and fixed in formalin-propionic acid-alcohol (FPA). Following this, the meristems were processed through a dehydration series of increasing ethanol solutions (70%, 90%, 95%, 100% w/safranin, and 100%). Following dehydration, the meristems were transferred into xylene followed by paraffin oil and, lastly, two changes in pure paraffin embedding wax. The tissues were carefully oriented and embedded in paraffin wax using a histo-embedder (Leica EG1160, Wetzlar, Germany). Meristem samples were sectioned to 8 µm using a microtome (Leica RM 2135, Germany). The slides were then stained using the Sharman stain series [26]. Slides were observed using a Laborlux D compound microscope (Leitz, Wetzlar, Germany). Images were captured using the Olympus DP73 microscope camera attachment (Leitz, Wetzlar, Germany). 

### 2.2. Scanning Electron Micrography

For each time point described above, meristems were dissected and fixed in formalin-acetic acid-alcohol (FAA). The meristems were dehydrated in increasing ethanol concentrations (70%, 90%, 95%, and 100%). The samples were dried using a critical point dryer (Polaron E3000, Hertfordshire, England). Following this, the samples were sputter coated using the 108 Auto Sputter Coater (Ted Pella, INC., Redding, CA, USA) with a gold target. The gold coated meristems were imaged using scanning electron micrography (SEM) (Hitachi SU3500, Hitachi High Technologies America, Inc., Pleasanton, CA, USA)

### 2.3. Sample Preparation for RNA-seq

RNA was extracted from dissected meristems for four data points, W0, W4, W6, and WB (early inflorescences) using the RNAeasy kit (Qiagen, Germantown, MD, USA). Each biological replicate for all timepoints (W0, W4, W6, and WB) is an apical meristem sample from one plant. The RNA samples were checked for quality using nanodrop one (Thermo Scientific, Madison, WI, USA), qubit (Thermo Scientific), and bioanalyzer (Agilent Technologies, Palo Alto, CA, USA)

### 2.4. RNA-seq Library Preparation and Sequencing

The RNA libraries were prepared using NEB Next Ultra II Directional RNA Library Prep Kit at University of California Riverside (UCR) genomics core. The RNA libraries were analyzed by the bioanalyzer. Low quality libraries were removed from the samples. At least three biological replicates for each data point were sequenced. A total of 17 libraries (3 each for W0 and W6, 6 for W4, and 5 for WB Table S1) were pooled and sequenced using a 1 × 75 × 6 cycle NextSeq v2 high output run. A sample tree was created using Spearman correlation. Biological replicates from the same time points clustered together and all time points had their own branch, which confirms the reproducibility of our results (Figure S1). The raw reads data was deposited to the NCBI’s Sequence Read Archive (SRA) accession # SRP218030, and BioProject # PRJNA559688.

### 2.5. Reference-Based Transcriptome Analysis

Reference-based transcriptome analysis was performed using our in-house R pipeline. The data analysis was conducted by implementing practices and methodology reviewed by Conesa et al. 2016 [27]. Raw reads were cleaned by removing the adapter, poly-N, and low-quality reads. Further analysis was performed on cleaned high quality reads. *Aquilegia coerulea* v3.1 genome was downloaded from Phytozome (https://phytozome.jgi.doe.gov/pz/portal.html) used as a reference to map the individual reads using HISAT2, which is a fast and sensitive alignment tool for mapping next-generation sequencing reads [28]. The read count was performed in a non-strand-specific way for exonic gene areas while ignoring overlaps between different genes. Subsequently, the expression count values were normalized by reads per kilobase per million mapped reads (RPKM). For each sample, reads overlapping with regions of interest were counted using the overlap function in R [29]. Sample-wise Spearman correlation coefficients from the transformed expression values were calculated using DESeq2 [30] and hierarchical clustering was performed with the hclust function in R (in stats package) and the results were plotted as a dendrogram.

### 2.6. Differentially Expressed Genes and Functional Enrichment Analysis 

The glm method of edgeR package was used for differential gene expression (DEG) analysis [31] and significant DEGs were filtered with a false discovery rate (FDR) of ≤0.05. A K-means clustering algorithm [32] was used to cluster transcripts showing differential co-expression at the sample level. The GO annotation of the reference-based transcriptome was performed using Blast2GO program [33]. The clusterProfiler was used for GO and KEGG enrichment analysis [34]. Over-represented GO terms were identified using a hypergeometric test with a significance threshold of 0.05 after Benjamini and Hochberg FDR correction [35]. 

## 3. Results and Discussions

### 3.1. Vernalization-Induced Developmental Changes in the Aquilegia SAM 

**Figure 2:**
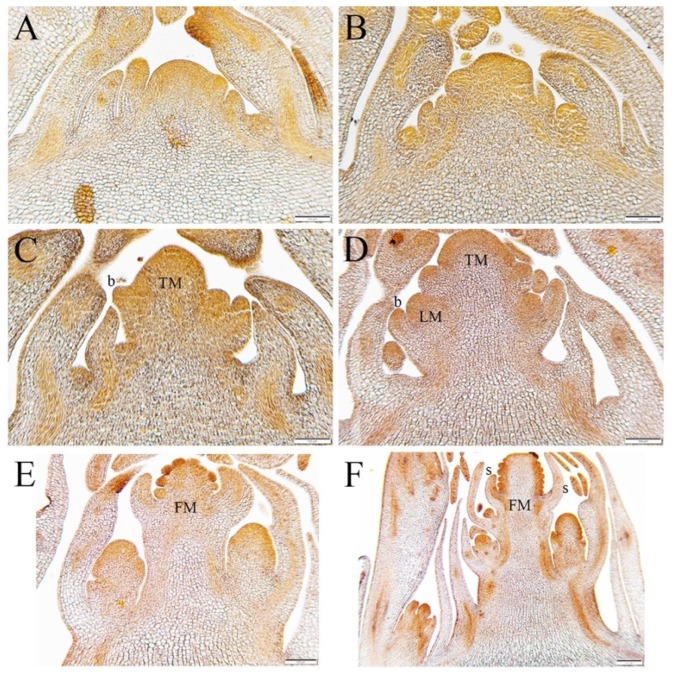
Developmental changes in meristem over the course of vernalization and post-vernalization. (**A**) Pre-vernalization W0. The SAM is vegetative. (**B**) W3 during vernalization. The apex is beginning to elongate. (**C**) Apex on the last day of vernalization (week 4). Elongation is continuing, the apical meristem is beginning to resemble a FM. (**D**) One-week post-vernalization. Inflorescence growth has progressed with the formation of new axillary meristems. (**E**) Two weeks post-vernalization, W6. Floral organ primordia can be seen in the terminal bud, which has clearly transitioned to the FM identity. (**F**) Four-weeks post vernalization, W8. A fully differentiated inflorescence with axillary meristems that are beginning to transition to the FM identity. Size bars A–E = 100 mm F = 200 mm. The symbol b = bract, s = sepal, TM = terminal meristem, LM = lateral meristem and, FM = floral meristem, organ primordia.

**Figure 3:**
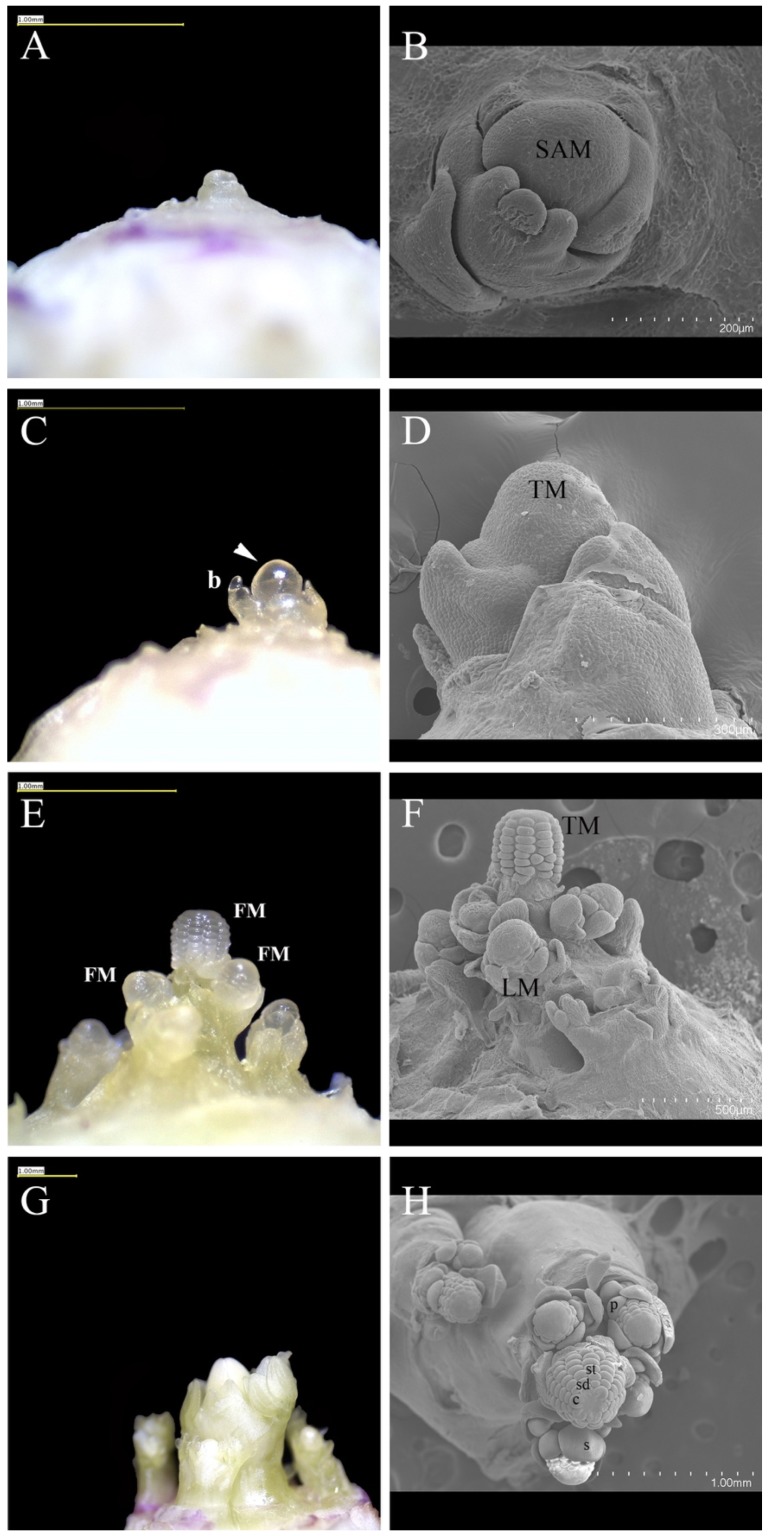
Changes in the apex observed through the stereoscope, A, C, E, G, and SEM, B, D, F, H, over the course of vernalization. (**A**,**B**) W0, SAM is vegetative. (**C**,**D**) W4 terminal meristem with subtending bracts. (**E**,**F**) W6 differentiating inflorescence. Floral organ primordia are visible in the terminal bud and several axillary meristems. (**G**,**H**) W8. The floral buds are fully formed and the compact inflorescence is prepared to bolt. The meristem remains vegetative. Size bars A, C, E, G, and H = 1.00 mm, B = 200 mm, D = 300 mm, and F = 500 mm. The symbol b = bract, s = sepal, p = petal, st = stamen, sd = staminodium, c = carpel, SAM = shoot apical meristem, FM = floral meristem, TM = terminal meristem, and LM = lateral meristem.

We conducted morphological studies at the macroscopic and microscopic level to analyze changes in the SAM over the course of the floral transition. *Aquilegia* is a rosette-forming perennial herb and, throughout development, the SAM is protected by sheathing leaf bases of the rosette leaves [19,36]. The SAM of 5–7 plants were dissected each week of the treatment and prepared for histological analysis (Figure 2) and SEM (Figure 3). Nine time points were sampled: pre-vernalization (week 0 or W0), each of the four weeks during vernalization (weeks 1–4 or W1–W4), and each of the four weeks post-vernalization (weeks 5–8 or W5–W8). Observations of the dissected shoot apex at week 0 revealed a vegetative SAM surrounded by spirally arranged compound leaves (Figure 2A and Figure 3A,B). This morphology was conserved through the first and second weeks of vernalization at 6 °C.

Morphological changes begin to be observed during the third week of vernalization (Figure 2B). At the end of week 3, the elongated SAM appears to be producing more lateral organs with active axillary meristems, which suggests a transition to the IM identity. At week 4, the terminal meristem continues to elongate, internodes appear to be forming, and lateral organs are less complex than the vegetative leaves, which are all consistent with the differentiation of the early inflorescence (Figure 2C and Figure 3C,D). After the first week post-vernalization (W5), the meristems showed the further differentiation of the inflorescence axis with discrete axillary meristems subtended by bracts (Figure 2D). After the second week of post-vernalization (W6), the terminal meristem has converted to floral identity and has initiated sepals, petals, and outer stamens (Figure 2E and Figure 3E,F). Initiation of floral organ primordia can also be seen in some lateral meristems (Figure 3F).

At W8, the fourth week of post-vernalization, we observe a fully developed primary inflorescence containing the terminal and the developing lateral floral meristems (Figure 2F and Figure 3G,H). Soon after, these plants bolted and the inflorescences emerged from the rosettes (Figure 1B). At this time point, shoot apices of non-vernalized control plants kept at 24 °C were dissected for comparison with their vernalized siblings. Observation under the dissecting microscope showed no IM development. 

This study in *A. coerulea* ‘Origami’ demonstrates that developmental changes in the SAM occur when the plant has been vernalized for 3 weeks, with more dramatic changes observed at week 4. This is a much shorter vernalization requirement compared to other previously studied North American species *A. formosa*. Most likely, this reduced requirement is the product of horticultural selection and hybridization in the generation of the ‘Origami’ lines, but it does provide a potential tool for future dissection of genetic differences between ‘Origami’ and species with longer vernalization requirements. In addition, it gives us a framework on which to base our developmental transcriptomic study.

### 3.2. Transcriptome Sequencing

**Figure 4:**
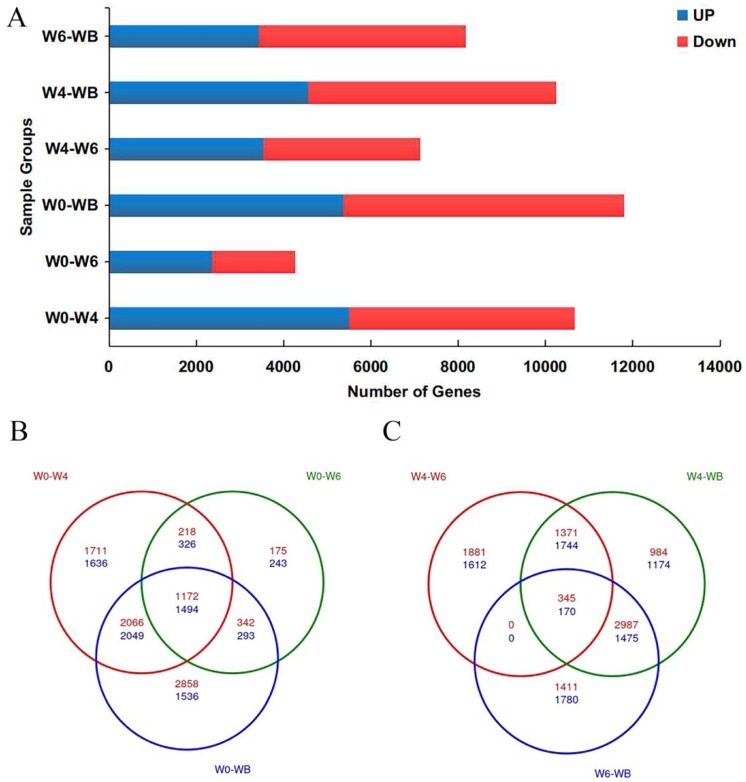
(**A**) Bar graph shows the number of genes up-regulated and down-regulated in different pairwise comparisons. (**B**) A three-way Venn diagram of DEGs in W0–W4, W0–W6, and W0–WB pairwise comparisons. (**C**) A three-way Venn diagram of DEGs in in W4–W6, W4–WB, and W6–WB pairwise comparisons.

We dissected apical meristems from plants at weeks 0, 4, 6, and B, with at least 3 biological replicates of each time point (Supplementary Table S1). Over 300 million reads were mapped to *Aquilegia’s* genome, with 89.5% successfully mapping and carried further for analysis of differential gene expression. We performed pairwise comparisons between W0–W4, W0–W6, W0–WB, W4–W6, W4–WB, W6, and WB (Figure 4A). The numbers of up-regulated and down-regulated genes in each pairwise comparison are listed in Supplementary Table S2. As expected, the W0–WB pairwise comparison of vegetative and fully elaborated inflorescences shows the greatest change in gene expression with 5372 up-regulated and 6432 down-regulated loci. However, even in the W0–W4 pairwise comparison, we observed a large number of differentially expressed genes, which suggested that, although the meristem is only slightly changed morphologically, the molecular program promoting flowering has been initiated. This is also very well shown by the Venn diagrams, where the specific vs. commonly up-regulated and down-regulated DEG’s in different pairwise comparisons are parsed out. In the first Venn diagram, we analyzed specific and commonly up-regulated genes between W0–W4, W0–W6, and W0–WB (Figure 4B) and, in the second, we analyzed W4–W6, W4–WB, and W6–WB (Figure 4C). Commonly up-regulated and down-regulated genes in the first comparison is 1172 and 1494, respectively, and, in the second, 345 and 170, respectively. Thus, the Venn diagram analysis agrees with the developmental results clearly showing the major change in gene expression is between W0–W4 (Figure 4B).

### 3.3. GO Enrichment and KEGG Analysis

To understand the functional classification of the DEGs in all of the pairwise comparisons, we assigned them GO terms. This classified the DEGs into three GO categories: biological process, molecular function, and cellular component. The most enriched GO terms are listed in Table 1, and the top 15 GO terms in each pairwise comparison are shown in Supplementary Figures S2–S4. Overall, these terms are consistent with the fact that these tissues are meristematic or early differentiating primordia with strong signals of transcriptional regulation, active metabolism, and protein processing. Involvement of DEGs in biological pathways was examined by performing KEGG analysis using *Arabidopsis thaliana* as the reference. We have used scatter plots to show the significant KEGG enrichment in all pairwise comparisons (Supplementary Figure S5). The enriched pathways are very consistent with the GO results.

### 3.4. Gene Expression Clusters

**Figure 5:**
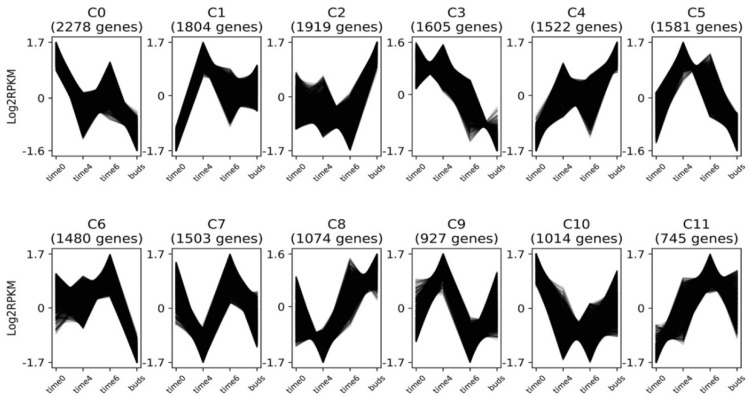
Clusters of co-expressed DEGs, C0–C11, at 4 time points W0 = time 0, W4 = time 4, W6 = time 6, and WB = buds.

**Figure 6:**
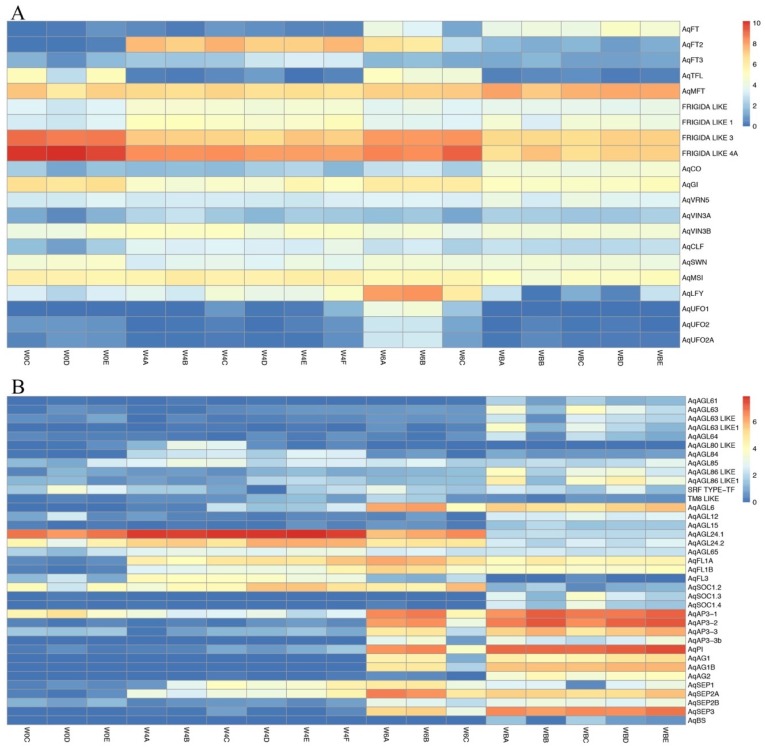
Heat map based of log 2 RPKM values. All the biological replicates used for each data-point are shown here. (**A**) Flowering time genes. (**B**) MADS-box genes.

*Clust* was used to generate 12 clusters, C0–C11, which shows co-expression of DEGs (Figure 5). The number of genes in each cluster range from 745 in Cluster 11 to 2278 in Cluster 0. We have analyzed the results by dividing them into two categories: (1) Flowering time genes (Table 2) and (2) MADS-box genes (Table 3). Heat maps generated for both the categories show their expression patterns across different data points in all biological replicates (Figure 6). We did not average the RPKM values for all biological replicates from one time point. Instead, we showed the expression pattern on the individual 17 samples to reflect the consistency and reproducibility of our results.

#### 3.4.1. Flowering Time Genes

The phosphatidylethanolamine-binding proteins (PEBPs) are known to play an important function in flowering time and inflorescence development. The family has three major clades [37,38], *FT*-like, *TERMINAL FLOWER*, or *TFL*-like, and *MOTHER OF FT* or *MFT*-like, all of which have homologs in *Aquilegia*. While *FT* induces flowering, *TFL* is known to delay flowering and promote indeterminacy in racemose inflorescences [39]. One *Aquilegia FT* homolog has been previously identified [25], *AqFT* (Aqcoe2G432300), but since the genome sequence has been completed (http://www.phytozome.net/), we have been able to identify two additional *FT* homologs: *AqFT2* (Aqcoe4G263600) and *AqFT3* (Aqcoe4G257600). The three paralogs of *AqFT* are in different clusters. *AqFT* is in C8 and shows strong up-regulation well after the floral transition in W6-WB. *AqFT2* is placed in C5 and shows the strongest expression coinciding with the floral transition itself. *AqFT3* is in C3 and appears to be expressed at low levels overall but does decrease following the floral transition. The other members of the PEBP family, *AqTFL* and *AqMFT*, fall into clusters 0 and 8, respectively. These diverse expression patterns indicate that the loci may have evolved different functional roles. By comparison, in sugar beet, two *FT* homologs have opposite functions such that *BvFT2* is a floral promoter and *BvFT1* is a floral repressor [17]. Similarly in *Nicotiana tabacum NtFT1*, *NtFT2*, and *NtFT3* are floral repressors while *NtFL4* is a floral promotor [38]. Exploration of the functions of the three *Aquilegia* paralogs may require the use of transgenics, but can also be performed in the context of natural variation that exists among species.

Another important flowering time gene is *FRIGIDA*. In *Arabidopsis FRIGIDA* is known for promoting *FLC*, which, in turn, keeps *FT* in a repressed state [40]. The *FRIGIDA* ortholog in *Aquilegia*, *AqFRI*, is rather ubiquitously expressed and does not fall in any cluster. We identified four other members of *FRI* family in *Aquilegia,* termed *AqFRI-like, AqFRI-like1, AqFRI-like3*, and *AqFRI-like4A.* The former two fall in cluster C9 and the latter two fall in cluster C0, which have largely opposing expression profiles. In Arabidopsis, *FRIGIDA-like 1 (FRL1)*, and *FRIGIDA-like 2 (FRL2)* have been shown to promote the winter annual habit in cooperation with *FRIGIDA* [41]. However, these functions converge on the regulation of *FLC*, which is absent from the *Aquilegia* genome. It remains possible that the more general biochemical function of FRI-like proteins, which is acting as a scaffold in the formation of transcriptional activation complexes [42], could be targeting other loci in *Aquilegia*.

The *GIGANTEA* and *CONSTANS* homologs are mostly known for promoting flowering in the context of the photoperiod pathway in *Arabidopsis*. A study that examined the physical interaction of the GI protein with the *FT* promoter suggested that GI alone or in a complex binds to the *FT* promoter to regulate *FT* expression in a *CO*-independent manner. This binding of GI to *FT* can possibly regulate the access of floral repressors such as *SVP* to the *FT* promoter [43]. The transcript level of *AqGI* is high in W0 and then decreases slightly in W4 followed by increased expression at W6, which places it in cluster C0. In *Aquilegia*, there are three *FT* homologs and they all have different expression profiles. It remains to be determined how AqGI is interacting with each of the *AqFT* paralogs to regulate flowering time. In this study, we see that *AqCO* spikes only at the WB stage, as shown in cluster *C2*. Analogous to the *FRI*-like loci described above, these genes are members of large gene families and their homologs have been shown to contribute to broad aspects of the photoperiod and circadian regulation [44,45,46]. However, it does not appear that *AqCO* is specifically up-regulated in response to the floral transition at W4, which could be consistent with the lack of the photoperiod response observed in other species of *Aquilegia* [25].

As with all major life cycle transitions, members of the Polycomb Repressor Complex 2 (PRC2) and their associated epigenetic complexes are critical to the floral transition in multiple model systems [47,48,49]. In *Aquilegia,* the three previously characterized PHD family members [50], *AqVRN5*, *AqVIN3A*, and *AqVIN3B,* fell into clusters C10, C1, and C9, respectively, while the three PRC2 members *AqCLF*, *AqSWN*, and *AqMSI* were found in clusters C1, C7, and C3, respectively. These patterns of differential expression suggest that epigenetic reprogramming may clear an important role in both promoting flowering and FM identity, consistent with what has been observed in Arabidopsis and other systems [51,52]. In particular, the strong induction of *AqCLF* coincident with W4 is intriguing given CLF’s known role in regulating floral MADS box genes [53].

Lastly, *LEAFY* (*LFY*) and *UNUSUAL FLORAL ORGANS (UFO)* homologs in *Arabidopsis* are known to be involved in the establishment of the floral meristem identity [54]. Expression of the three *UFO* homologs, *AqUFO1* and *AqUFO2 AqUFO2A*, and single *AqLFY* homolog peak at the week 6 stage. *AqLFY*, *AqUFO2*, and *AqUFO2A* are co-expressed in cluster C6 while *AqUF01* is in C11. These patterns are consistent with more detailed studies that have found expression to be limited to the earliest stages of floral development [Sharma et al. 2019, in press]. 

#### 3.4.2. MADS-Box Genes

The MADS-box gene family is pan-eukaryotic but is very well-studied in plants, where members are critically involved in many aspects of development, particularly flowering time and floral organ identity [55,56]. MADS-box genes are broadly classified as type I and type II, but all MADS-box genes encode a highly conserved DNA-binding domain that consists of ~58–60 amino acids [57,58]. In the *A. thaliana* genome, there are approximately 100 annotated MADS-box genes, of which about 40 are type II. In this study, we found 43 MADS-box genes expressed in the four sampled stages (W0, W4, W6, WB), but only 37 of these were differentially expressed between stages (Figure 6B). Assigning these loci to specific gene lineages reveals that multiple MADS-box genes have duplicated paralogs [56].

Several of the type II MADS-box genes are known to influence flowering time or the early stages of inflorescence development. The most notable of these is *FLC*, which is absent from *Aquilegia*. Another floral repressor is the *SHORT VEGETATIVE PHASE* (*SVP*), but this gene lineage is specific to the core eudicots [25] and, therefore, also missing from *Aquilegia*. The *SVP* paralog *AGL24*, in contrast, is a promoter of flowering [12]. In *Aquilegia*, two representatives of this subfamily were named *AqAGL24.1* and *AqAGL24.2*, and the latter was found to be strongly expressed in IMs [25]. In the current dataset, we again see different expression profiles between paralogs, with *AqAGL24.1* most strongly expressed in the earliest stages of the floral transition while *AqAGL24.2* appears to be shifted later. This is consistent with the published *in situ* data for *AqAGL24.2*, which was strongly expressed in IMs, but functional studies are necessary to understand whether the paralogs are redundant or possibly even opposing in function. Previous studies in *Aquilegia* identified additional potential IM identity loci, which are homologs of the *AP1/FUL* gene lineage [36]. *AqFL1A/B* are expressed in axillary meristems and, when they are silenced, plants produce less complex inflorescences, which suggests that they cannot maintain IM identity [36]. In our analysis, we see a very low expression of both genes in the W0 vegetative meristem, but expression is very strong at stages W4 and W6 when IMs form.

Collectively, homologs of the floral organ identity genes are up-regulated at stages W6 or WB, consistent with their previously characterized expression patterns [23,24,25,59]. One interesting difference is that the putative stamen and carpel identity genes *AqAG1* and *AqAG2* are in different clusters (Table 3), with *AqAG2* coming up later than *AqAG1*, which is in line with its stronger expression in the late arising carpels [24,59,60]. The so-called E class genes, which act as transcriptional activators in conjunction with the other floral organ identity genes, are represented by four homologs in *Aquilegia*: *AqSEP1*, the tandem duplicates *AqSEP2A* and *AqSEP2B*, and *AqSEP3* [56]. *AqSEP1* and *AqSEP2A* are activated immediately after vernalization in W4 whereas the *AqSEP2B* and *AqSEP3* are expressed at W6, which potentially reflects some differentiation among their functions. *AqAGL6* is closely related to the *SEP* lineage [61,62] and its homolog in *Nigella* has been shown to primarily control sepal identity [63]. *AqAGL6* expression peaks at W6 and persists into W8, which suggests potential redundancy with the *SEP* homologs in floral organ identity, but requires further study.

Expression of the detected type I MADS-box genes, which commonly function in gametophyte development [64,65], is generally induced following vernalization. However, the tissues sampled are not late enough to include gametophytic tissues. Therefore, these detected loci are interesting candidates for alternative functions in earlier stages of floral development.

## 4. Conclusions

This study has demonstrated that *Aquilegia coerulea* ‘Origami’ initiates detectable developmental changes in the SAM by the third week into vernalization and four weeks of treatment are sufficient to promote the full floral transition. This is considerably shorter than the previously studied *A. formosa* and *A. vulgaris*, which have longer requirements of five to six weeks [25,50]. Using this developmental information as a guide for transcriptomics has provided a dataset that can now be mined for promising candidate genes that may act as either floral repressors or activators. Our preliminary analysis of the results already reveals several potential floral repressors, including paralogs of *FT* and *AGL24*. By combining this resource with QTL analyses of species with differing requirements for vernalization such as *A. formosa* and ‘Origami’, we can explore the genetic basis of vernalization in this distinct evolutionary lineage that achieves the response without previously characterized loci such as *FLC*. Our ability to ultimately manipulate this trait in diverse plant lineages depends on our understanding of the many independent derivations of the vernalization response.

## Figures and Tables

**Table 1:** Top GO terms in the three categories: Biological Process (BP), Molecular Function (MF), and Cellular Components (CC) in all pairwise comparisons.

Pairwise Comparisons	GO Category	GO Term	Description
W0-W4 up	BP	GO:0070647	Protein modification by small protein conjugation or removal
W0-W6 up	BP	GO:0010033	Response to organic substance
W0-WB up	BP	GO:0070647	Protein modification by small protein conjugation or removal
W4-W6 up	BP	GO:0055086	Nucleobase-containing small molecule metabolic process
W4-WB up	BP	GO:0034660	ncRNA metabolic process
W6-WB up	BP	GO:0048856	Anatomical structure development
W0-W4 down	BP	GO:0055086	Nucleobase-containing small molecule metabolic process
W0-W6 down	BP	GO:0006091	Generation of precursor metabolites and energy
W0-WB down	BP	GO:0006091	Generation of precursor metabolites and energy
W4-W6 down	BP	GO:0006091	Generation of precursor metabolites and energy
W4-WB down	BP	GO:0006812	Cation transport
W6-WB down	BP	GO:0006091	Generation of precursor metabolites and energy
W0-W4 up	MF	GO:0003700	DNA-binding transcription factor activity
W0-W6 up	MF	GO:0003700	DNA-binding transcription factor activity
W0-WB up	MF	GO:0003700	DNA-binding transcription factor activity
W4-W6 up	MF	GO:0005198	Structural molecule activity
W4-WB up	MF	GO:0005198	Structural molecule activity
W6-WB up	MF	GO:0003700	DNA-binding transcription factor activity
W0-W4 down	MF	GO:0005198	Structural molecule activity
W0-W6 down	MF	GO:0005198	Structural molecule activity
W0-WB down	MF	GO:0005198	Structural molecule activity
W4-W6 down	MF	GO:0003700	DNA-binding transcription factor activity
W4-WB down	MF	GO:0015075	Ion transmembrane transporter activity
W6-WB down	MF	GO:0015075	Ion transmembrane transporter activity
W0-W4 up	CC	GO:0031974	Membrane-enclosed lumen
		GO:0043233	Organelle lumen
		GO:0070013	Intracellular organelle lumen
W0-W6 up	CC	GO:0005829	Cytosol
W0-WB up	CC	GO:0031974	Membrane-enclosed lumen
		GO:0043233	Organelle lumen
		GO:0070013	Intracellular organelle lumen
W4-W6 up	CC	GO:0005840	Ribosome
W4-WB up	CC	GO:0005840	Ribosome
W6-WB up	CC	GO:0031974	Membrane-enclosed lumen
		GO:0043233	Organelle lumen
		GO:0070013	Intracellular organelle lumen
W0-W4 down	CC	GO:0005840	Ribosome
W0-W6 down	CC	GO:0005840	Ribosome
W0-WB down	CC	GO:0098796	Membrane protein complex
W4-W6 down	CC	GO:0009579	Thylakoid
		GO:0044434	Chloroplast part
		GO:0044435	Plastid part
W4-WB down	CC	GO:0044435	Plastid part
W6-WB down	CC	GO:0098796	Membrane protein complex

**Table 2:** Cluster number of flowering time genes.

Flowering Time Genes	Cluster Number
*AqTFL*	C0
*FRIGIDA LIKE 3*	C0
*FRIGIDA LIKE 4A*	C0
*AqGI*	C0
*AqVIN3A*	C1
*AqCLF*	C1
*AqCO*	C2
*AqFT3*	C3
*AqMSI*	C3
*AqFT2*	C5
*AqLFY*	C6
*AqUFO2*	C6
*AqUFO2A*	C6
*AqSWN*	C7
*AqFT*	C8
*AqMFT*	C8
*FRIGIDA LIKE*	C9
*FRIGIDA LIKE 1*	C9
*AqVIN3B*	C9
*AqVRN5*	C10
*AqUFO1*	C11

**Table 3:** Cluster number of MADS-box genes.

MADS-Box TFs	Cluster Number
*AqAGL61*	C2
*AqAGL63*	C2
*AaAGL63 LIKE*	C2
*AqAGL64*	C2
*AqAGL86 LIKE*	C2
*AqAGL86 LIKE A*	C2
*AqAGl12*	C2
*AqSOC1.3*	C2
*AqSOC 1.4*	C2
*AqAG1*	C2
*AqBS*	C2
*AqAGL24.1*	C3
*AqAGL80 LIKE*	C3
*AqAG2*	C4
*AqAGL84*	C5
*AqAGL85*	C5
*AqAGL24.2*	C5
*AqAGL65*	C5
*TM8 LIKE*	C6
*AqSOC1.2*	C6
*AqAGL15*	C8
*AqAP3-1*	C8
*AqAP3-2*	C8
*AqAP3-3*	C8
*AqAG1B*	C8
*AqSEP2B*	C8
*AqSEP3*	C8
*AqAGL63 LIKE A*	C8
*AqFL3*	C9
*SRF TYPE-TF*	C10
*AqAGL6*	C11
*AqFL1A*	C11
*AqFL1B*	C11
*AqAP3-3b*	C11
*AqPI*	C11
*AqSEP1*	C11
*AqSEP2A*	C11

## Supplementary Materials

The Supplementary Material for this article are available online at https://www.mdpi.com/2073-4425/10/10/734/s1. Figure S1: The clustering of all 17 samples at four timepoints done using the Spearman correlation. All biological replicates for one time point cluster as a separate group, Figure S2: Top 15 GO terms in Biological Process category in all pairwise comparison, Figure S3: Top 15 GO terms in Molecular Function category in all pairwise comparison, Figure S4: Top 15 GO terms in Cellular Component category in all pairwise comparison, Figure S5: Scatter plot of KEGG pathway enrichment in all pairwise comparisons. Table S1: The alignment statistics result for all samples. Table S2: Number of genes up and down regulated in different pairwise comparisons.
