# Supplementary material for: Developmental and Molecular Changes Underlying the Vernalization-Induced Transition to Flowering in Aquilegia coerulea (James)
Source: Genes (Basel). 2019 Sep 22;10(10):734. doi: 10.3390/genes10100734 (PMC6826667; doi:10.3390/genes10100734)
Supplement: Supplementary file 1 [file genes-10-00734-s001.docx]

**Supplementary Materials**


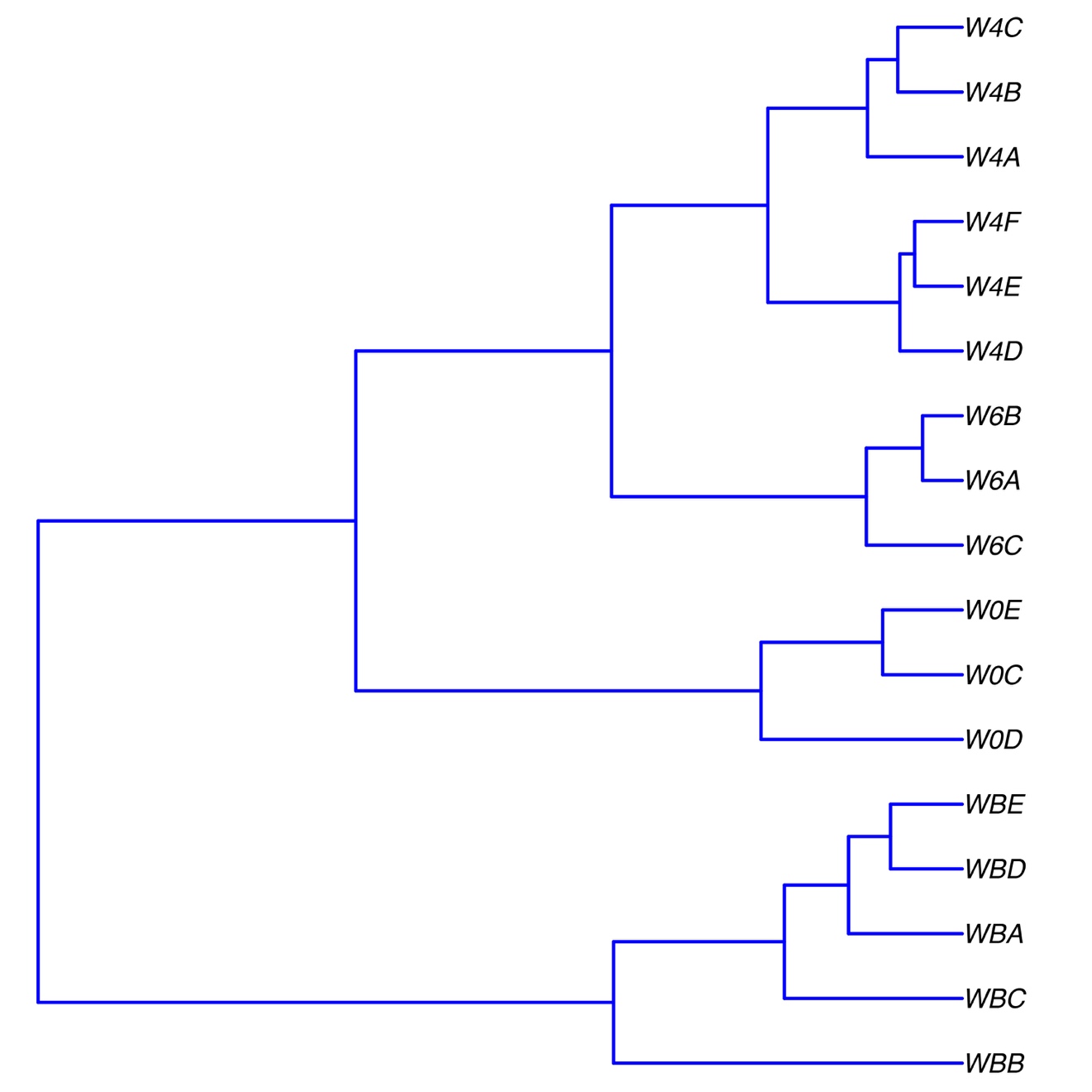


**Figure S1.** The clustering of all 17 samples at 4 timepoints done using the Spearman correlation. All biological replicates for one time point cluster as a separate a group.

**
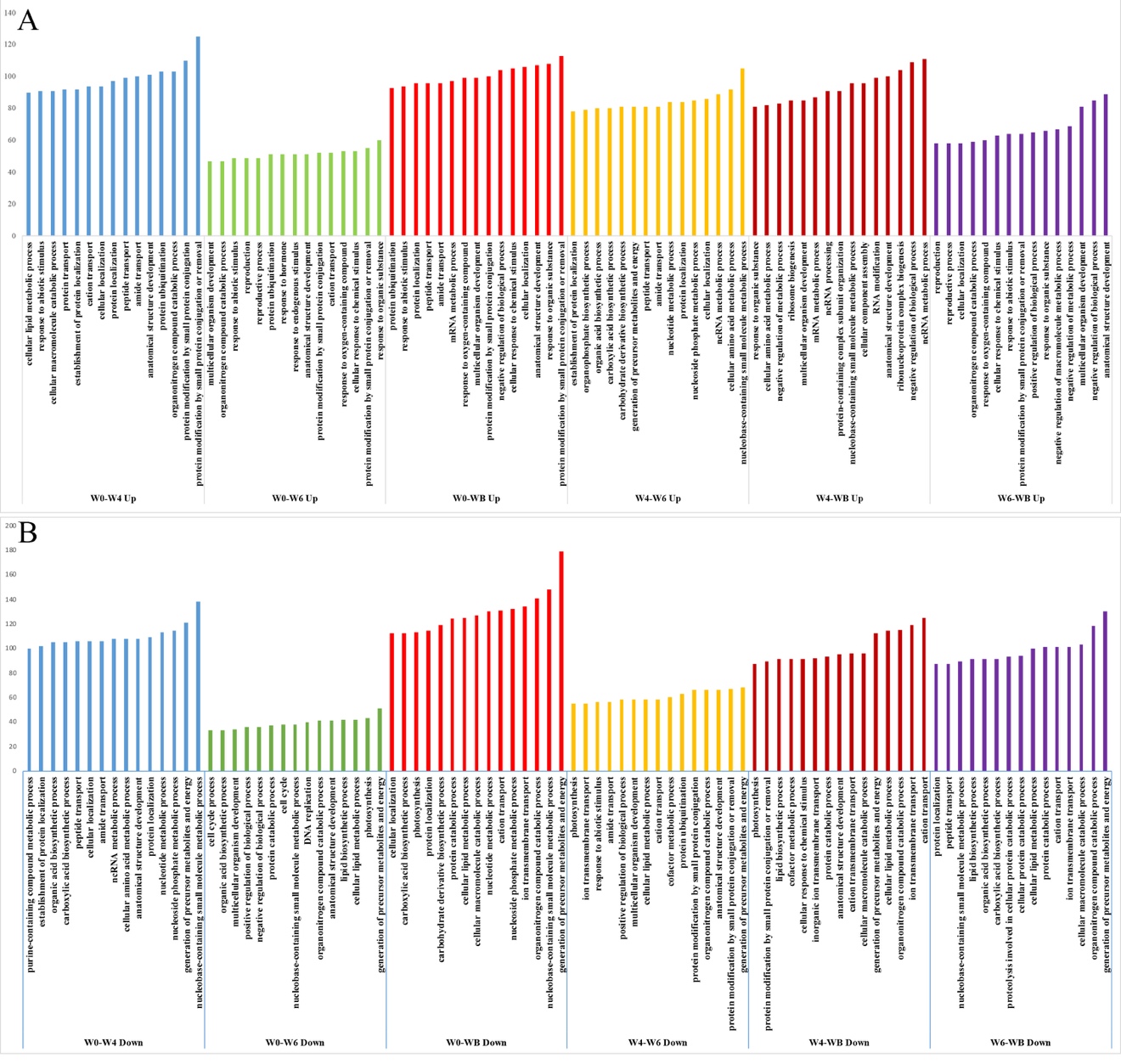
**

**Figure S2.** Top 15 GO terms in Biological Process category in all pairwise comparison.


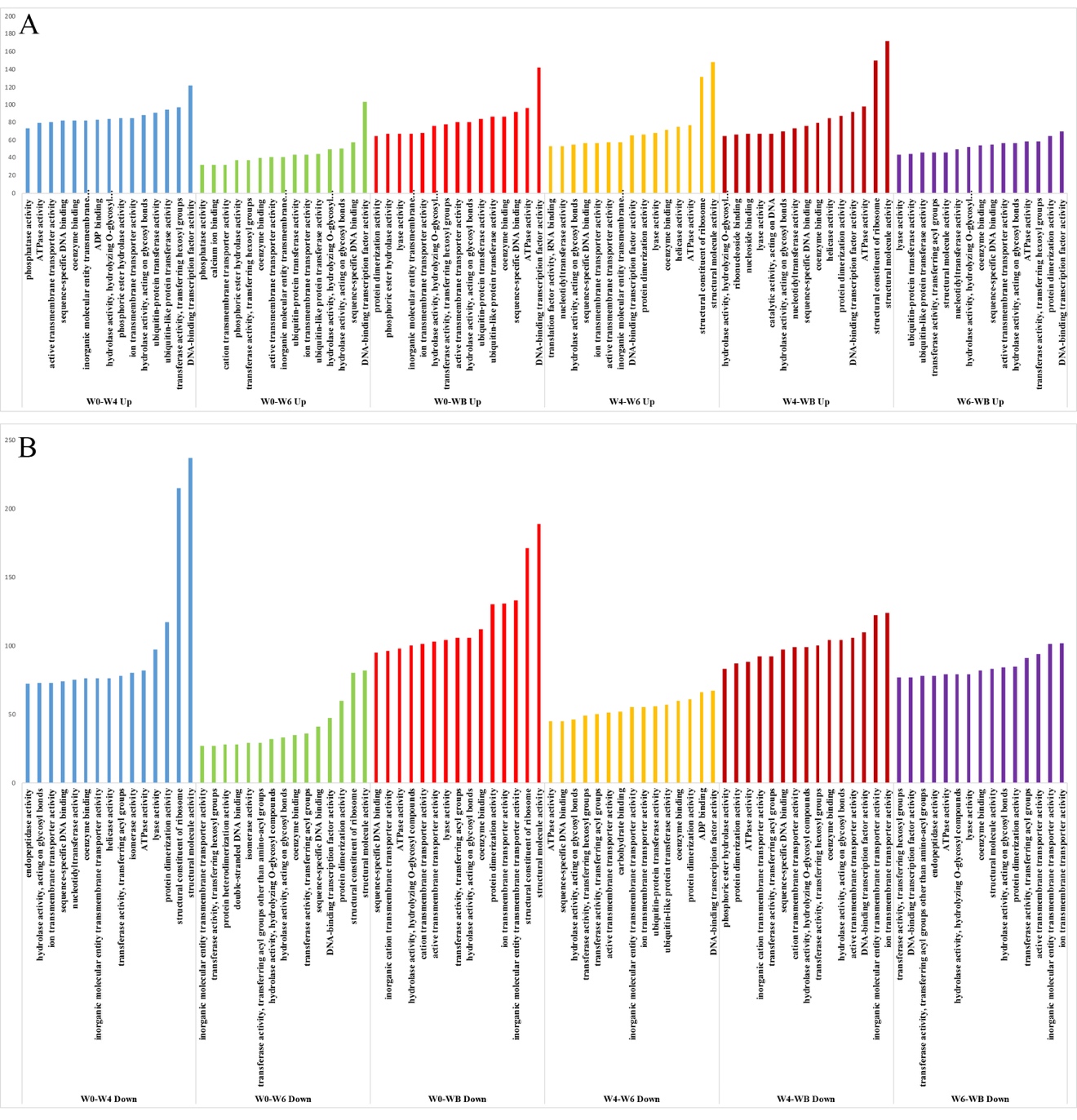


**Figure S3.** Top 15 GO terms in Molecular Function category in all pairwise comparison.


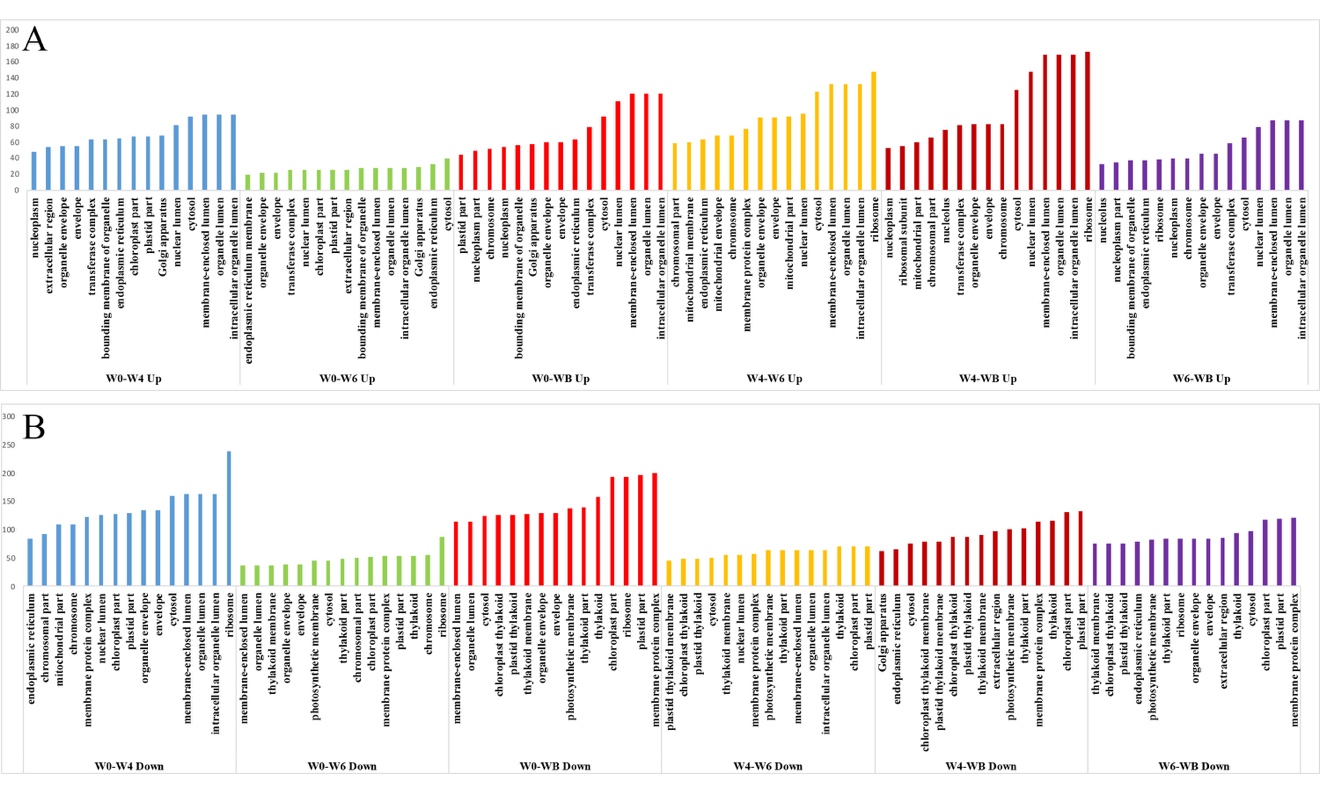


**Figure S4.** Top 15 GO terms in Cellular Component category in all pairwise comparison.


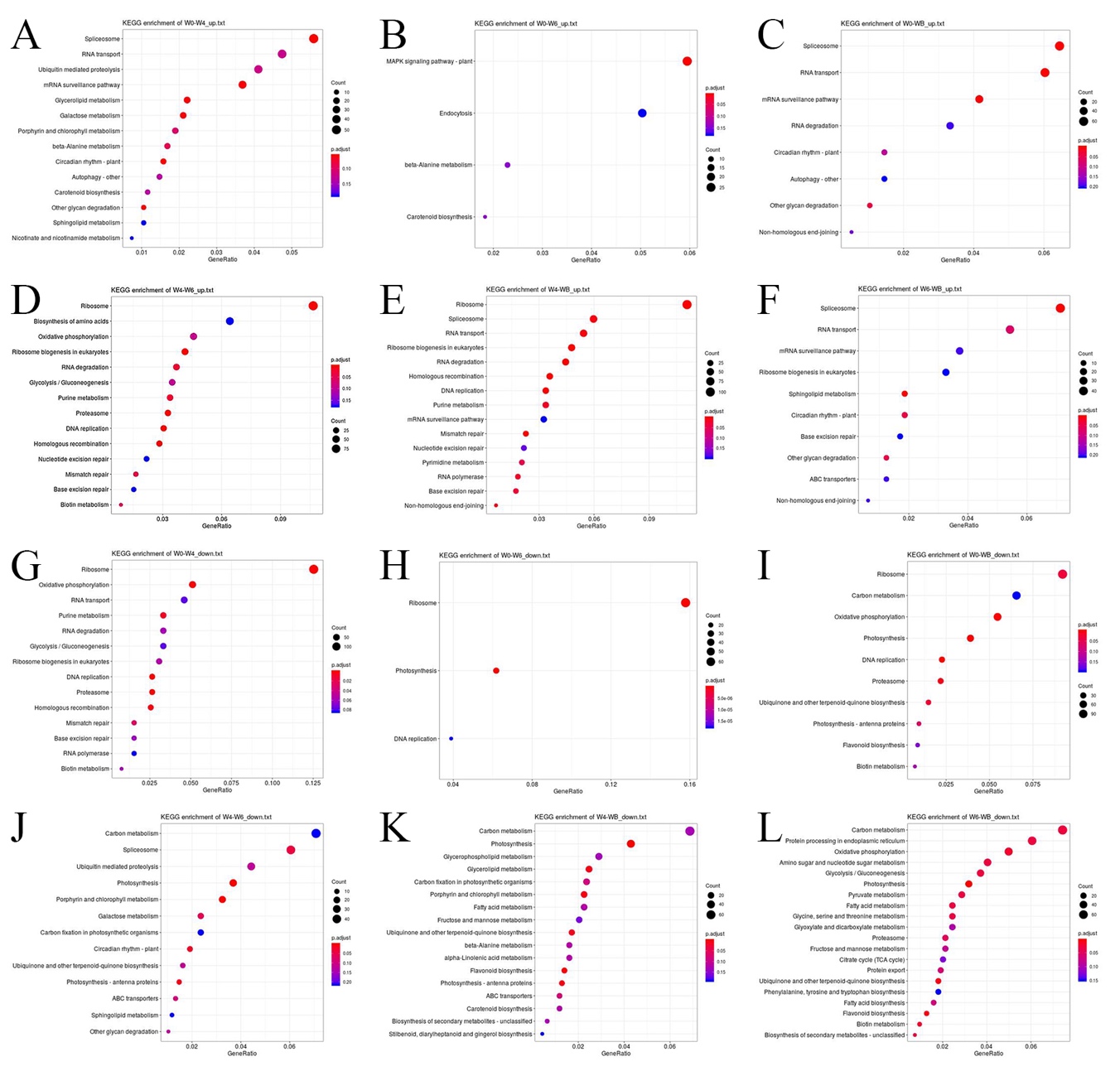


**Figure S5.** Scatter plot of KEGG pathway enrichment in all pairwise comparisons.

**Table S1.** The alignment statistics result for all samples.

| **Library** | **File Name** | **Number-reads** | **Number-align** | **Percentage- Aligned** |
| --- | --- | --- | --- | --- |
| 1 | W0C | 17213605 | 16259642 | 94.4580871 |
| 2 | W0D | 16868959 | 15694597 | 93.0383256 |
| 3 | W0E | 19539369 | 18639622 | 95.3952095 |
| 4 | W4A | 19570939 | 18564229 | 94.8560976 |
| 5 | W4B | 22720453 | 21655907 | 95.3145917 |
| 6 | W4C | 19125681 | 13981533 | 73.1034518 |
| 7 | W4D | 18208916 | 16072586 | 88.2676706 |
| 8 | W4E | 17657008 | 15002147 | 84.9642646 |
| 9 | W4F | 19272656 | 17942515 | 93.0982995 |
| 10 | W6A | 21143814 | 19268598 | 91.1311365 |
| 11 | W6B | 17423968 | 16448444 | 94.4012523 |
| 12 | W6C | 21664555 | 20032770 | 92.4679505 |
| 13 | WBA | 19055832 | 13424673 | 70.449157 |
| 14 | WBB | 19698498 | 15884785 | 80.6395747 |
| 15 | WBC | 16367793 | 15206110 | 92.902629 |
| 16 | WBD | 17688768 | 16645541 | 94.1023196 |
| 17 | WBE | 15185346 | 14197313 | 93.4935101 |

**Table S2.** Number of genes up and down regulated in different pairwise comparisons.

| **Pairwise comparisons** |  | **UP** | **Down** |
| --- | --- | --- | --- |
| W0-W4 |  | 5505 | 5167 |
| W0-W6 |  | 2356 | 1907 |
| W0-WB |  | 5372 | 6438 |
| W4-W6 |  | 3526 | 3597 |
| W4-WB |  | 4563 | 5687 |
| W6-WB |  | 3425 | 4743 |
